# 477. Characteristics and Long-Term Outcomes of Cardiac Complications of SARS-CoV-2 in Youth: Results from the EPICC YES C3 Prospective Longitudinal Cohort Module

**DOI:** 10.1093/ofid/ofad500.547

**Published:** 2023-11-27

**Authors:** Milissa U Jones, Stephanie A Richard, Allison M Malloy, Kirk N Liesemer, Rebecca Sainato, Joseph May, Brian Hughes, Rhonda E Colombo, Nikhil Huprikar, David Saunders, David Lindholm, Katrin Mende, Christina Schofield, Anuradha Ganesan, Erik R Johnson, Charles T Nguyen, Zachary S Turner, Mark P Simons, David Tribble, Robert O’Connell, Brian Agan, Timothy Burgess, Patrick W Hickey, Craig Dobson, Simon Pollett, Ryan Flanagan

**Affiliations:** Uniformed Services University, Bethesda, Maryland; Infectious Disease Clinical Research Program, Department of Preventive Medicine and Biostatistics, Uniformed Services University of the Health Sciences, Bethesda, MD, USA, Bethesda, Maryland; Department of Pediatrics, Uniformed Services University of the Health Sciences, Bethesda, MD, USA, Bethesda, Maryland; Madigan Army Medical Center, Tacoma, Washington; Madigan Army Medical Center, Tacoma, Washington; Walter Reed National Military medical Center, Bethesda, Maryland; Uniformed Services University, Bethesda, Maryland; Infectious Disease Clinical Research Program, USUHS; Henry M. Jackson Foundation for the Advancement of Military Medicine, Inc., Bethesda, Maryland; Walter Reed National Military Medical Center, Bethesda, Maryland; Uniformed Services University of the Health Sciences, Bethesda, MD, USA, Bethesda, Maryland; Department of Medicine, Uniformed Services University of the Health Sciences; Brooke Army Medical Center, San Antonio, Texas; 1Infectious Disease Clinical Research Program, Department of Preventive Medicine and Biostatistics, Uniformed Services University of the Health Sciences and Brooke Army Medical Center, JBSA Fort Sam Houston, TX, San Antonio, TX; Madigan Army Medical Center, Tacoma, Washington; Infectious Disease Clinical Research Program, Bethesda, Maryland; Tripler Army Medical Center, Honolulu, Hawaii; Tripler Army Medical Center, Honolulu, Hawaii; Brooke Army Medical Center, San Antonio, Texas; Infectious Disease Clinical Research Program, Department of Preventive Medicine and Biostatistics, Uniformed Services University of the Health Sciences, Bethesda, MD, USA, Bethesda, Maryland; Infectious Disease Clinical Research Program, Department of Preventive Medicine and Biostatistics, Uniformed Services University of the Health Sciences, Bethesda, MD, USA, Bethesda, Maryland; Infectious Disease Clinical Research Program, USUHS, Bethesda, Maryland; Infectious Disease Clinical Research Program, Department of Preventive Medicine and Biostatistics, Uniformed Services University of the Health Sciences, Bethesda, MD, USA, Bethesda, Maryland; Infectious Disease Clinical Research Program, Department of Preventive Medicine and Biostatistics, Uniformed Services University of the Health Sciences, Bethesda, MD, USA, Bethesda, Maryland; Uniformed Services University, Bethesda, Maryland; Children's Hospital of Philadelphia, Philadelphia, Pennsylvania; Infectious Disease Clinical Research Program, Department of Preventive Medicine and Biostatistics, Uniformed Services University of the Health Sciences, Bethesda, MD, USA, Bethesda, Maryland; Tripler Army Medical Center, Honolulu, Hawaii

## Abstract

**Background:**

Few studies have described cardiac sequela of SARS-CoV-2 infection in children across the spectrum of acute COVID-19 disease severity, particularly milder outpatient cases without multisystem inflammatory syndrome in children (MIS-C). We sought to define the frequency and long term outcomes of coronary artery aneurysms, depressed cardiac function, and other cardiac complications among youth positive for SARS-CoV-2.

**Methods:**

EPICC is a multisite, prospective, COVID-19 cohort study of Military Health System (MHS) beneficiaries. Participants eligible for the Youth Echo Screening for Cardiac Complications of COVID (YES-C3) module were SARS-CoV-2 positive, age 0-22 years and without prior history of coronary aneurysm or autoimmune disease. Participants were scheduled for echocardiography (echo) and 12 lead electrocardiogram (ECG) at enrollment and four weeks after enrollment. Those with abnormal findings had repeated echo and ECG studies at 6 and/or 12 months; clinical data were abstracted from the medical record.

**Results:**

Between January 2021 and March 2022, 154 participants were enrolled in the YES C3 module, with median age of 10.7 years (IQR 6.4- 17.2) (Table 1). 32%, 45%, and 23% experienced their first COVID-19 infection in the pre-Delta, Delta, and Omicron era, respectively. The majority of those enrolled did not require hospitalization, with only 2 of 154 (1.3%) hospitalized, both with MIS-C. 19% of participants were fully vaccinated at the time of their infection. Among 129 participants with an echo or an ECG, 4 (3.1% (95% CI 0.09%-7.7%)) had abnormalities attributed to COVID 19 (Table 2), including 1 MIS-C case. By 6-month follow up, all echo abnormalities had resolved without intervention and no deaths were noted out to 12 months post enrollment.

**Figure:**
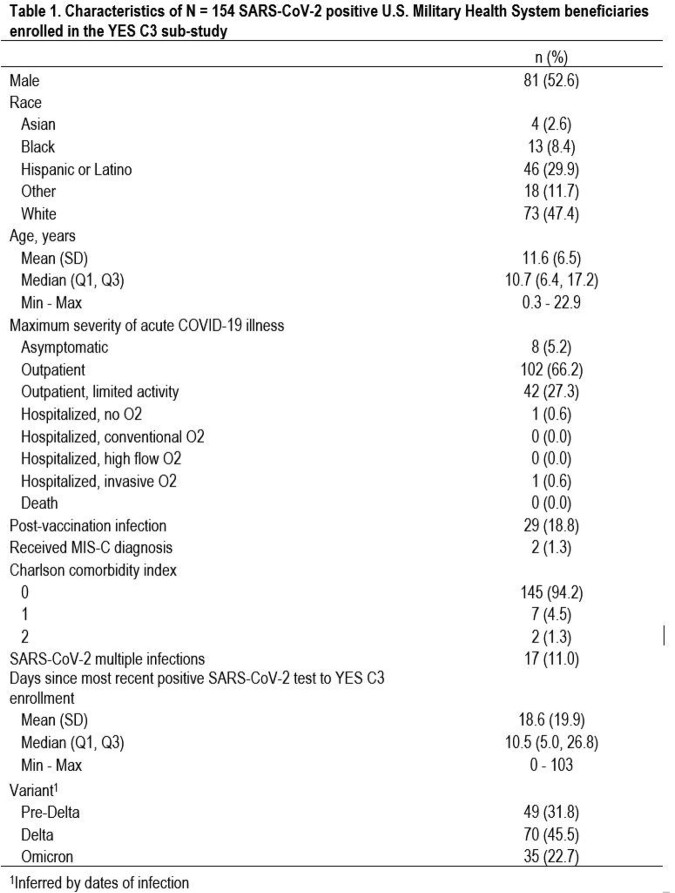

**Figure:**
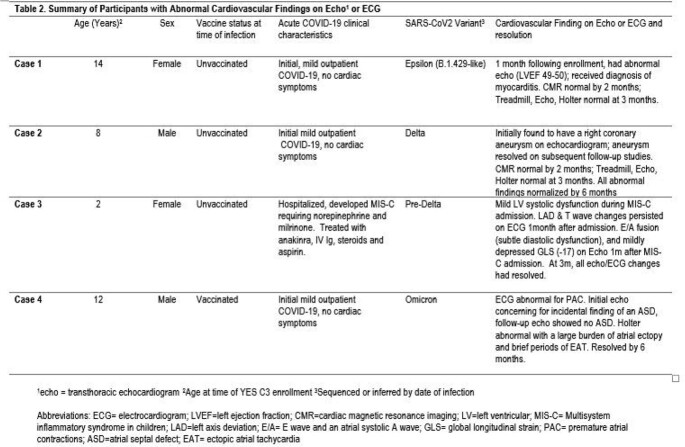

**Conclusion:**

We observed few cardiovascular complications among YES C3 participants who generally experienced mild infection, with most enrolled in the pre-vaccine and pre-Omicron era. The frequency of MIS-C and cardiac abnormalities detected may reflect tertiary medical center enrollments. All echo abnormalities resolved within 3-6 months. Our findings support current guidelines which advise against routine screening echo or ECGs for young persons with mild SARS-CoV-2 infection without cardiac symptoms.

**Disclosures:**

**Mark P. Simons, PhD**, AstraZeneca: The IDCRP and HJF were funded to conduct an unrelated phase III COVID-19 monoclonal antibody immunoprophylaxis trial as part of US Govt COVID Response **David Tribble, MD, DrPH**, AstraZeneca: The IDCRP and HJF were funded to conduct an unrelated phase III COVID-19 monoclonal antibody immunoprophylaxis trial as part of US Govt COVID response **Timothy Burgess, MD, MPH**, AstraZeneca: The IDCRP and the Henry M. Jackson Foundation (HJF) were funded to conduct an unrelated phase III COVID-19 monoclonal antibody immunoprophylaxis trial **Simon Pollett, MBBS**, AstraZeneca: The IDCRP and the Henry M. Jackson Foundation (HJF) were funded to conduct an unrelated phase III COVID-19 monoclonal antibody immunoprophylaxis trial

